# Comparison of efficacy and side effects between short-course radiotherapy and chemotherapy with or without immunotherapy in the neoadjuvant treatment for patients with locally advanced rectal cancer

**DOI:** 10.3389/fonc.2025.1630570

**Published:** 2025-09-29

**Authors:** Shaoqing Niu, Jie Wen, Yangchan Li, Jiayi Yang, Jianqi Xiong, Zhuangzhuang Yang, Chuangqi Chen, Yunying Yang, Yong Bao

**Affiliations:** ^1^ Department of Radiation Oncology, The First Affiliated Hospital, Sun Yat-sen University, Guangzhou, China; ^2^ Department of Interventional Oncology, The First Affiliated Hospital, Sun Yat-sen University, Guangzhou, China; ^3^ Gastrointestinal Surgery Center, The First Affiliated Hospital, Sun Yat-sen University, Guangzhou, China

**Keywords:** locally advanced rectal cancer, neoadjuvant therapy, short-course radiotherapy, immunotherapy, complete response

## Abstract

**Purpose:**

To evaluate the efficacy and safety of short-course radiotherapy (SCRT) combined with chemotherapy (ChT) with or without immunotherapy (IT) in the neoadjuvant treatment for patients with locally advanced rectal cancer (LARC).

**Materials and methods:**

Clinicopathological data were retrospectively collected from LARC patients treated with SCRT combined with ChT with or without IT at our cancer center, from July 2021 to November 2024. The SCRT dose was 25 Gray (Gy), delivered daily for five consecutive days per week. The neo-ChT regimens included CapeOx and mFOLFOX. The IT regimen included cadonilimab, toripalimab, tislelizumab and sintilimab. The primary endpoint was the complete response rate after neoadjuvant treatment, including clinical complete response (cCR) and pathological complete response (pCR) rates. According to the treatment method, patients were divided into two groups: patients who received neoadjuvant SCRT and ChT with IT (SCRT+IT) group and who received neoadjuvant SCRT and ChT without IT (SCRT) group. The chi-square test was used to compare CR rates between groups, and logistic regression models were employed for univariate and multivariate analyses.

**Results:**

From July 2021 to November 2024, of 50 LARC patients undergoing SCRT at our institution, 41 met the inclusion criteria: SCRT+IT group: (n=20) and SCRT group (n=21). Following neoadjuvant treatment, the SCRT+IT group achieved a complete response (CR) rate of 65.0% (13/20), comprising 9 pCR and 4 cCR. The SCRT group exhibited a pCR rate of 19.0% (4/21). This difference in CR rates was statistically significant (*p* = 0.003). Univariate and multivariate logistic regression analyses identified treatment modality (SCRT+IT *vs*. SCRT) as the sole independent predictor of CR (univariate: *p* = 0.003; multivariate: *p* = 0.017). The most common adverse reactions were grades 0 – 2, such as myelosuppression and radiation-induced rectal injury (RRI). 3 patients developed postoperative anastomotic leakage. In the SCRT+IT cohort, one patient experienced grade 3 immune-mediated hepatitis and one patient developed grade 3 immune-related pneumonitis.

**Conclusion:**

Compared to SCRT combined with ChT alone, the combination of SCRT with ChT and IT significantly improves CR rate after neoadjuvant treatment, and the treatment-related adverse effects were tolerable.

## 1 Introduction

Colorectal cancer (CRC) ranks in third place in terms of incidence and second in terms of mortality according to global cancer statistics for the year 2022 (1). According to the statistical results, over 50% of CRC patients in China are diagnosed with advanced-stage cancer at the time of initial diagnosis (2). The results of previous studies showed that neoadjuvant treatment is playing an increasingly important role for patients with locally advanced rectal cancer (LARC) (3–5). Both long-course radiotherapy (LCRT) and short-course radiotherapy (SCRT), along with chemotherapy (ChT), constitute the main modalities of neoadjuvant therapy (6). Furthermore, LARC patients who achieve a clinical complete response (cCR) to neoadjuvant therapy could avoid surgery and opt for a “watch-and-wait” management strategy (3).

Previous studies have reported that the CR rates of patients who received neo-CRT vary from 12%–28% (4, 7, 8). Emerging preclinical studies demonstrated that hypo-fractionated radiotherapy (RT) may have stronger immunostimulatory effects than conventional fractionation, indicating the potential for combining hypo-fractionated RT with immunotherapy (IT) to synergistically enhance tumor cell killing (9, 10). Recently, several randomized trials reported that SCRT combined with IT regimens led to promising pCR rates in patients with LARC. In UNION clinical trial, 113 patients with LARC patients in the experimental arm who received neoadjuvant SCRT followed by camrelizumab plus CAPOX and the results showed that the pCR rates were 39.8% which was significantly higher than that of the control arm (11). In TORCH clinical trial, 121 LARC patients with proficient mismatch repair or microsatellite stable (pMMR/MSS) who received SCRT followed by six cycles of consolidation ChT and IT (capecitabine and oxaliplatin and toripalimab) or two cycles of induction ChT and IT followed by SCRT and the rest four doses were enrolled, and the CR rates of two groups were 56.5% and 54.2% respectively (12).

To further explore and comp the CR rates of LARC patients were as high as 39.8%–56.5% in the UNION and are the efficacy and toxicity between neoadjuvant SCRT and ChT with or without IT in patients with LARC, we conducted this retrospective study. We hope that the findings of this study will offer evidence for the selection of treatment protocols in clinical practice and contribute to further enhancing the therapeutic efficacy for patients with LARC.

## 2 Materials and methods

### 2.1 Patient selection

In this study, we retrospectively collected the clinical data of LACR patients who admitted to our cancer center from July 2021 to November 2024. Clinical pathological data, including sex, age, tumor site, tumor-node-metastasis (TNM) stage pathological result and treatment methods, were collected.

This study was a retrospective investigation and the clinicopathological data were collected from the medical record and the results of examination. This study was approved by our institutional medical ethics committee with Ethical Approval No [2025]. 404. Informed consent were obtained from all patients.

The inclusion criteria were as follows: (1) patients were aged between 18 and 80 year; (2) histologically confirmed adenocarcinoma; (3) primary tumor located in the rectum; (4) stage II to III disease as according to the 8th edition of the Union for American Joint Cancer Committee (AJCC) TNM staging system; (5) received neoadjuvant SCRT and ChT, either with or without IT. The exclusion criteria encompassed: (1) a history of other malignant tumors; (2) uncontrolled medical conditions such as heart failure or psychiatric disorders; (3) pregnant female patients.

### 2.2 Details of RT

All patients received SCRT. RT was delivered using volumetric modulated arc therapy (VMAT). The gross tumor volume (GTV) was defined as the primary tumor (GTVp) and positive lymph nodes (GTVn). The clinical target volume (CTV) included the GTV plus areas at risk for microscopic spread from the primary tumor and mesorectum and the lymphatic drainage area of the anterior sacrum and internal iliac region (13).

A 5 mm isotropic expansion of the CTV was used to create the planning target volume (PTV). The prescribed dose for the PTV was 25 Gray (Gy), delivered daily for five consecutive days per week. RT was delivered via a modern linear accelerator delivering a 6 MV photon beam, and treatment plans were calculated using the Monaco^®^ (Elekta AB, Stockholm, Sweden) system or Eclipse^®^ (Varian Medical Systems, Inc, Palo Alto, CA, USA).

### 2.3 ChT and IT regimens

The neo-ChT regimens included (1) CapeOx: oxaliplatin 130 mg/m² on day 1 + capecitabine 1000 mg/m² twice daily on days 1–14, every 3 weeks; (2) mFOLFOX: oxaliplatin 85 mg/m² on day 1 + calcium levofolinate 200 mg/m² on day 1 + 5-Fu 400 mg/m² bolus on day 1, followed by 2400 mg/m² continuous infusion for 46–48 hours, every 2 weeks. The median number of cycles of neoadjuvant ChT was 3 (range: 1–9).

The IT regimen included cadonilimab (10mg/kg, every 3 weeks), toripalimab (240 mg, every 3 weeks), tislelizumab (200 mg, every 3 weeks), and sintilimab (200 mg, every 3 weeks). The median number of cycles of IT was 3 (range: 2–4).

During RT, neither concurrent ChT nor immunotherapy IT was administered.

### 2.4 Follow-up and data analysis

Survival follow-up was performed using telephone or clinic visits every three months. The primary endpoints were CR rates, including cCR and pathological CR (pCR). cCR was defined as the absence of residual disease at 8–12 weeks after neoadjuvant therapy and during the subsequent follow-up period, and reassessment methods included DRE, endoscopy and/or pathological examination, and pelvic hr-MRI. pCR was defined as no residual tumor cells in the resected tumor tissue and regional lymph nodes after surgery. The secondary endpoints were overall survival (OS), recurrence-free survival (RFS), distant metastasis-free survival (DMFS) and side effects. Continuous data were recorded using medians and interquartile ranges (IQRs), whereas categorical data were recorded using numbers and percentages (%). Survival analyses were performed via log-rank analysis using SPSS software (version 29.0), and a *P* value < 0.05 was considered statistically significant.

## 3 Results

### 3.1 Clinicopathological characteristics

**Figure 1:**
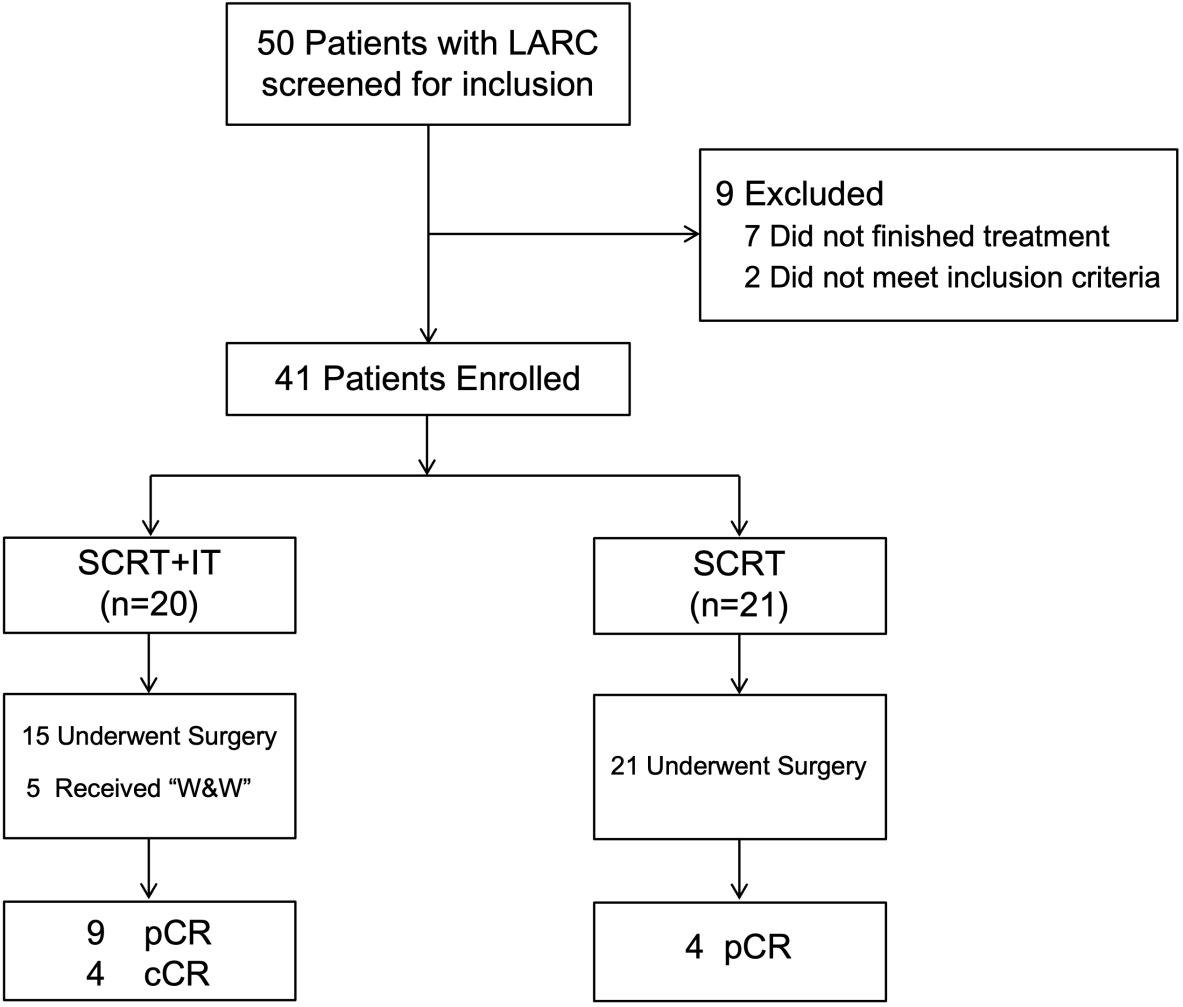
CONSORT diagram of the patients in this study.

A total of 50 patients with LARC who were diagnosed and received neoadjuvant SCRT and ChT with or without IT at our cancer center from July 2021 to November 2024, and 41 patients who met the inclusion criteria were included in this study. A consort diagram of the study is shown in **Figure 1**.

The median age was 58 years (range, 22–79 years), and 31 patients (75.6%) were male. There were 8 patients (19.5%) with stage II disease and 33 patients (80.5%) with stage III disease. At the time of diagnosis, 18 patients (43.9%) presented with elevated carcinoembryonic antigen (CEA) levels, and 7 patients with elevated carbohydrate antigen 19-9 (CA199) levels. The mismatch repair (MMR) status of 35 patients was documented, including 34 patients (97.1%) were proficient mismatch repair (pMMR) and only one patient (2.9%) was deficient mismatch repair (dMMR). Regarding risk factors, 22 patients (53.7%) were diagnosed with positive mesorectal fascia (MRF) and 7 patients (17.1%) were extramural venous invasion (EMVI). The clinicopathological characteristics of the 41 patients are summarized in **Table 1**.

**Table 1:** Baseline clinical characteristics of the 41 patients with LARC.

Characteristic	Total	Groups		*P* value
	N (%)	SCRT+IT	SCRT	
Age (years)				
≥ 60	27 (65.9)	15 (75.0)	12 (57.1)	0.228
< 60	14 (34.1)	5 (25.0)	9 (42.9)	
Gender				
Male	31 (75.6)	15 (75.0)	16 (76.2)	0.929
Female	10 (24.4)	5 (25.0)	5 (23.8)	
Location of tumor				
Upper	3 (7.3)	2 (10.0)	1 (4.8)	0.497
Middle	18 (43.9)	7 (35.0)	11 (52.4)	
Lower	20 (48.8)	11 (55.0)	9 (42.9)	
Clinical stage (AJCC eighth ed.)				
II	8 (19.5)	4 (20.0)	4 (19.0)	0.939
III	33 (80.5)	16 (80.0)	17 (81.0)	
MRF				
Positive	22 (53.7)	9 (45.0)	13 (61.9)	0.278
Negative	19 (46.3)	11 (55.0)	8 (38.1)	
EMVI				
Positive	7 (17.1)	2 (10.0)	5 (23.8)	0.240
Negative	34 (82.9)	18 (90.0)	16 (76.2)	
Peritoneal reflection involved				
Yes	7 (17.1)	1 (5.0)	6 (28.6)	0.045
No	34 (82.9)	19 (95.0)	15 (71.4)	
CEA level				
Elevated	18 (43.9)	6 (30.0)	12 (57.1)	0.080
Noraml	23 (56.1)	14 (70.0)	9 (42.9)	
CA199 level				
Elevated	7 (17.1)	2 (10.0)	5 (23.8)	0.240
Normal	34 (82.9)	18 (90.0)	16 (76.2)	
MMR status^$^				
pMMR	34 (97.1)	18 (5.3)	16 (100.0)	0.367
dMMR	1 (2.9)	1 (94.7)	0	
Cycles of ChT				
1-3	25 (61.0)	18 (90.0)	7 (33.3)	< 0.001
> 3	16 (39.0)	2 (10.0)	14 (66.7)	
IT regimens				
Cadonilimab		16 (80.0)	–	
Toripalimab		2 (10.0)	–	
Sintilimab		1 (5.0)	–	
Tislelizumab		1 (5.0)	–	
Clinical outcome				
CR	17 (41.5)	13 (65.0)	4 (19.0)	0.003
no-CR	24 (58.5)	7 (35.0)	17 (81.0)	

SCRT, short-course radiotherapy; IT, immunotherapy; AJCC, American Joint Committee on Cancer; MRF, mesorectal fascia; EMVI, extramural venous invasion; CEA, carcinoembryonic antigen; CA199, carbohydrate antigen 19-9; MMR, mismatch repair; pMMR, proficient mismatch repair; dMMR, deficient mismatch repair; ChT, chemotherapy; CR, complete response.

The RT dose for all 41 patients was all 25Gy in 5 fractions. The most common neoadjuvant ChT regimen was CapeOx (n=38) and the rest patients received mFOLFOX regimen (n=3). The median number of cycles of neoadjuvant ChT was 3 (range: 1–9). A total of 20 patients received IT in the stage of neoadjuvant treatment, and the median number of cycles of IT was 3 (range: 2–4). Regarding to IT regimen, 16 patients received cadonilimab, 2 patients received toripalimab, one patient received tislelizumab, and one patient received sintilimab. The surgical procedures include intersphincteric resection (ISR, n=3), Dixon (n=25), Miles (n=7) and local excision (n=1).

### 3.2 CR rates of two groups

Among the 41 patients enrolled, 17 patients achieved CR, including 13 patients achieved pCR and 4 patients achieved cCR. The total CR rate was 41.5% (17/41) in the whole cohort.

**Figure 2:**
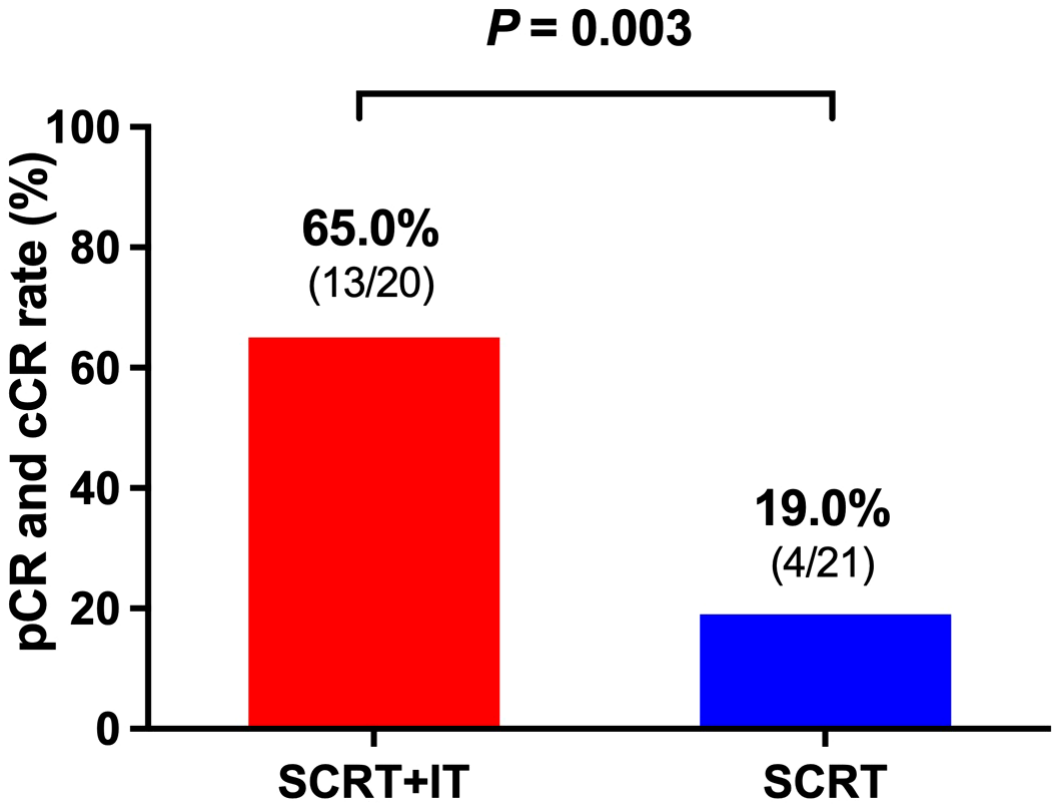
The CR rate of patients in SCRT+IT group was significantly higher than that of patients in SCRT group (*p* = 0.003). The CR rate of patients in SCRT+IT group was 65.0% (13/20) and the CR rate of patients in SCRT group was 19.0% (4/21). SCRT, short-course radiotherapy; IT, immunotherapy.

According to the treatment method, patients were divided into two groups: patients who received neoadjuvant SCRT and ChT with IT (SCRT+IT) group and who received neoadjuvant SCRT and ChT without IT (SCRT) group. The CR rate of patients in SCRT+IT group was 65.0% (13/20), including 9 patients achieved pCR and 4 patients achieved cCR. The CR rate of patients in SCRT group was 19.0% (4/21), including 4 patients achieved pCR. The CR rate of patients in SCRT+IT group was significantly higher than that of patients in SCRT group (*p* = 0.003, **Figure 2**).

### 3.3 Univariate and multivariate analysis

According to the univariate analysis, only treatment method (SCRT+IT *vs*. SCRT) was significantly associated with improved CR rate (*p* = 0.003).

Then, we incorporated the clinical pathologic and treatment-related factors into the multivariate analysis. The results of the multivariate analysis revealed that treatment method (SCRT+IT *vs*. SCRT) remained a solely independent prognostic factor for CR rate (**Table 2**).

**Table 2:** Univariate and multivariate analysis of CR rates of 41 LARC patients.

Variable	Univariate analysis			Multivariate analysis		
	HR	95% CI	*P* value	HR	95% CI	*P* value
Age (years) (≥ 60y vs. < 60y)	0.694	0.183-2.628	0.591	0.870	0.134-5.642	0.884
Gender (Male vs. Female)	0.923	0.216-3.945	0.914	0.843	0.119-5.983	0.864
Clinical Stage (Stage II vs. III)	0.343	0.069-1.694	0.178	0.218	0.026-1.832	0.161
MRF (Positive vs. Negative)	0.635	0.182-2.219	0.476	1.350	0.223-8.175	0.744
EMVI (Positive vs. Negative)	0.188	0.020-1.729	0.109	0.221	0.017-2.838	0.247
Cycles of neo-ChT (> 3 vs. < 3 cycles)	0.492	0.132-1.837	0.288	3.257	0.316-33.610	0.321
Treatment methods (SCRT+IT vs. SCRT)	0.789	1.898-32.817	0.003	18.495	1.676-204.145	0.017

CR, complete response; LARC, locaaly advanced rectal cancer; HR, hazard ratio; CI, confidence interval; MRF, mesorectal fascia; EMVI, extramural venous invasion; ChT, chemotherapy; SCRT, short-course radiotherapy; IT, immunotherapy.

### 3.4 Survival and failure pattern

**Figure 3:**
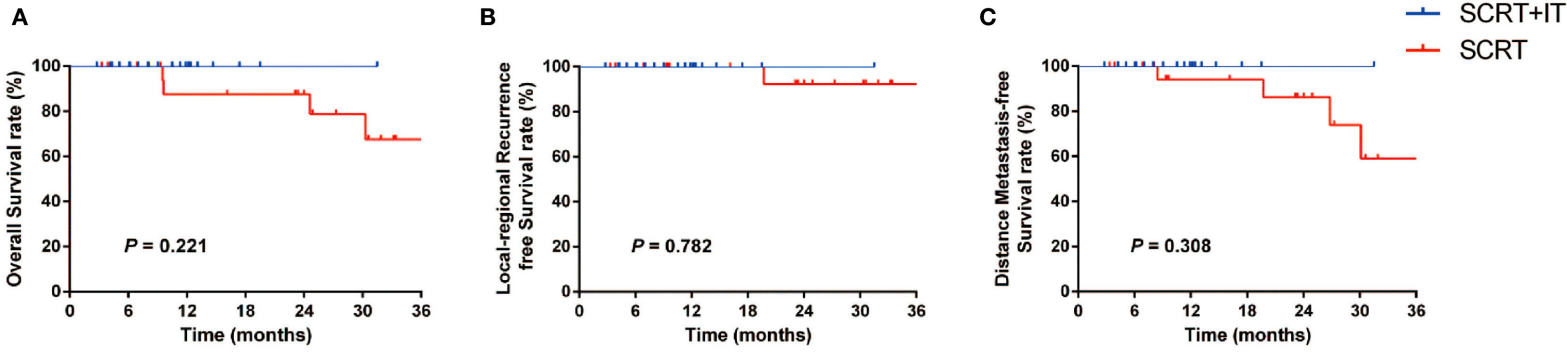
**(A-C)** Subgroup analysis of OS **(A)**, LRFS **(B)** and DMFS **(C)** according to the treatment method (SCRT+IT *vs*. SCRT). SCRT, short-course radiotherapy; IT, immunotherapy.

The median follow-up time was 12.4 months (range: 3.3–44.4 months). The predicted 1-year OS, LRFS and DMFS rates were 92.6%, 100% and 96.6%, respectively (**Figures 3A-C**). In subgroup analysis, the 1-year OS rates of patients in SCRT+IT and SCRT group were 100% and 87.5% (*p* = 0.221), 1-year LRFS rates were both 100% and 100% (*p* = 0.782), and 1-year DMFS rates were 100% and 94.1%, (*p* = 0.308), respectively.

During the follow-up period, one patient experienced local recurrence and distant metastases (DM). The sites of recurrence were the external iliac and inguinal lymphatic lymph nodes, and the DM site was lung. The other three patients developed DM only, and the sites of DM included the lung, bone and mediastinal lymph nodes.

### 3.5 Treatment-related side effects and management

All 41 patients finished treatment as planned without interruption. Acute toxicities were evaluated according to the Common Terminology Criteria for Adverse Events (CTCAE) version 5.0. The most common adverse reactions were grades 0–2, such as myelosuppression and radiation-induced rectal injury (RRI). Among the 20 patients who received SCRT+IT, one patient developed a grade III IT-related hepatitis, and one patient developed a grade III IT-related pneumonia, and both two patients recovered after receiving immunoglobulin and corticosteroids therapy. Regarding the postoperative adverse reactions, 3 patients in the SCRT group had anastomotic fistula and they all recovered after undergoing surgical treatment. The incidences of adverse reactions are summarized in **Table 3**.

**Table 3:** Acute toxicity during neoadjuvant treatment and surgery.

Treatment-related side effects	Groups by treatment			
	SCRT+IT		SCRT	
	n (%) of patients by grade		n (%) of patients by grade	
	1-2	3-4	1-2	3-4
Myelosuppression				
Leukopenia	13 (92.9)	1 (7.1)	16 (84.2)	3 (15.8)
Neutropenia	15 (100)	0 (0.0)	14 (87.5)	2 (12.5)
Thrombocytopenia	9 (45.0)	3 (15.0)	12 (80.0)	3 (20.0)
Anemia	12 (80.0)	3 (20.0)	13 (65.0)	7 (35.0)
RRI	6 (100.0)	0	5 (100.0)	0
IT-related adverse reactions				
Liver function abnormalities	0	1 (100.0)	–	–
Pneumonia	0	1 (100.0)	–	–
Complications of surgery^#^	Yes	No	Yes	No
Anastomotic leakage	0 (0.0)	15 (100.0)	3 (14.3)	18 (85.7)

SCRT, short-course radiotherapy; IT, immunotherapy; RRI, radiation−induced rectal injury.

**^#^** In the SCRT+IT group, 15 patients underwent surgery; in the SCRT group, 21 patients underwent surgery.

For myelosuppression, graded treatment were adopted: for grade 1 neutropenia, observe closely; for grade 2–3, administer G-CSF and pause anti-tumor therapy; for grade 4, pause anti-tumor therapy, administer G-CSF and broad-spectrum antibiotics (if febrile). Transfuse platelets for counts < 20×10^9^/L or active bleeding and red blood cells for severe anemia (Hb < 60g/L).

For the management of radiation-induced rectal injury, we often use loperamide or smectite for diarrhea, mesalazine (oral/suppository/enema) for mucosal inflammation or bleeding, and probiotics for gut dysbiosis. Adjust diet to low-fat, easily digestible foods; pause RT for grade 3 injury.

## 4 Discussion

The main finding of this study was that the combination of SCRT with IT could significantly improve CR rate of LARC patients after neoadjuvant treatment. This result was similar with previous studies (11, 12). The UNION clinical trial compared the efficacy and safety of SCRT followed by CapeOx and camrelizumab (experimental arm) and LCRT followed by CapeOx alone (control arm) as neoadjuvant treatment for patients with LARC, the results showed that the pCR rates were 39.8% in the experimental arm compared to 15.3% in the control arm (*p* < 0.001) (11). The results of TORCH clinical trial showed that the combination of programmed cell death-1 (PD-1) inhibitor with total neoadjuvant therapy (iTNT) could remarkably improve CR rates in LARC patients and the CR was achieved at 56.5% and 54.2% in two experimental groups (12).

In this study, the CR rate achieved 65.0% in the SCRT and IT group, which was higher than the above two clinical trials. And the results of univariate and multivariate also indicate that the addition of IT to SCRT was an independent prognostic factor for CR rate. The main reason may be the IT regimen adopted in different studies, the camrelizumab which was used in the UNION study and the toripalimab which was used in the TORCH study are both PD-1 inhibitor. However, most of the patients (16/20, 80.0%) who were in SCRT+IT group in this study received cadonilimab, which is a bispecific antibody targeting both PD-1 and cytotoxic T-lymphocyte-associated protein-4 (CTLA-4). Previous clinical studies have shown that the combination of anti-CTLA-4 antibodies and anti-PD-1 antibodies can significantly improve the outcomes of some refractory tumors (14–16). As the world’s first approved bispecific antibody targeting both PD-1 and CTLA-4, cadonilimab can block the interactions between PD-1 and CTLA-4 and their ligands PD-L1/PD-L2 and B7.1/B7.2 (17). By inhibiting PD-1, it can relieve the immunosuppression of T cells, and by inhibiting CTLA-4, it can promote the activation of tumor-specific T cell immunity, thus synergistically enhancing the anti-tumor therapeutic effect.

The results of previous studies showed that the use of IT has shown promising results in colorectal patients with dMMR tumors (18, 19). In this study, the only one patient with dMMR, who was diagnosed as T3N0M0 (stage II), received neoadjuvant treatment including SCRT with 3 cycles of IT (cadonilimab) and ChT (CapeOx). She achieved cCR after neoadjuvant treatment and chose the “W&W” strategy. At the time of last follow-up, she was still under cCR without recurrence or DM with the follow-up time of 17.4 months.

However, we should also notice that IT will also bring immune-related adverse events (irAEs). A meta-analysis enrolled 8730 patients who received PD-1 or PD-L1 inhibitors to analyze the incidence rate (IR) of irAEs, and the result showed that the IR of any grade irAEs is 17.1% and IR of grade ≥3 irAEs is 4.0% (20). So, when patients present irAEs, the timely recognition, evaluation and management were required, and corticosteroids and supportive care were needed in some severe situation (21). In this study, there were two patients in SCRT+IT group developed a grade III IT-related hepatitis and a grade III IT-related pneumonia, but both two patients recovered after receiving timely therapy and finished the treatment.

In this study, we employed an RT dose of 25 Gy delivered in 5 fractions, which was recommended by the NCCN guideline and widely used in clinical practice. However, there were several studies evaluated a simultaneous integrated boost (SIB) up to 30 Gy. Pollom et al. did a phase II trial (SHORT-FOX) which was presented in Poster Session in 2024 ASCO (No.3600), and patients with > T2N0 or low T2N0M0 rectal adenocarcinoma were enrolled. Patients underwent radiation (25 Gy/5 fractions + 5 Gy/1 fraction boost) followed by 8 cycles of FOLFOXIRI. Among the 37 patients, 9 (24.3%) patients achieved a cCR and 8 (21.6%) achieved a near cCR, and the adverse effect were mild.

There were several limitations in this study. First, the sample size was relatively small, and the follow-up time is relatively short, so the results need to be validated with lager samples and longer follow-up times. Second, this was a retrospective study, and the treatments such as the regimen of ChT and IT were not uniform, thus prospective randomized controlled trials are warranted to validate our findings. Thirdly, some clinical data was unavailable, such as MMR status and the expression of PD-L1 level.

In conclusion, compared with SCRT combined with ChT alone, the combination of SCRT with ChT and IT could significantly improve CR rate of LARC patients after neoadjuvant treatment, and the treatment-related adverse effects were tolerable.

## Data Availability

The raw data supporting the conclusions of this article will be made available by the authors, without undue reservation.
